# COVID-19 Vaccine Booster Hesitancy in Malaysia: A Web-Based Cross-Sectional Study

**DOI:** 10.3390/vaccines11030638

**Published:** 2023-03-13

**Authors:** Kai Wei Lee, Sook Fan Yap, Hooi Tin Ong, Myo Oo, Kye Mon Min Swe

**Affiliations:** 1 Department of Pre-Clinical Sciences, M. Kandiah Faculty of Medicine and Health Sciences, Universiti Tunku Abdul Rahman, Kajang 43000, Selangor, Malaysia; lee_kai_wei@yahoo.com (K.W.L.); onght@utar.edu.my (H.T.O.); 2 Centre for Research on Communicable Diseases, Universiti Tunku Abdul Rahman, Kajang 43000, Selangor, Malaysia; 3 Department of Medical Microbiology, Faculty of Medicine and Health Sciences, Universiti Putra Malaysia, Serdang 43400, Selangor, Malaysia; 4 Department of Population Medicine, M. Kandiah Faculty of Medicine and Health Sciences, Universiti Tunku Abdul Rahman, Kajang 43000, Selangor, Malaysia; myo@utar.edu.my; 5 Department of Medicine, M. Kandiah Faculty of Medicine and Health Sciences, Universiti Tunku Abdul Rahman, Kajang 43000, Selangor, Malaysia; kyemon@utar.edu.my

**Keywords:** COVID-19, immunization, vaccination, vaccine hesitancy, booster hesitancy

## Abstract

Vaccination is a key public health strategy that is known to be effective in mitigating the risk of infection and severe disease. However, in the context of the COVID-19 pandemic, the percentage (<50%) of Malaysians who have received a booster for the COVID-19 vaccine has remained stagnant over a year. This study aimed to determine the prevalence of and the factors associated with hesitancy toward the second dose of booster for the COVID-19 vaccine. A web-based cross-sectional study was conducted from August to November 2022. The Oxford Vaccine Hesitancy Scale was used to assess the hesitancy toward the second dose of booster for the COVID-19 vaccine. Simple and multiple factors logistic regressions were used to determine the predictors of hesitancy. A *p*-value less than 0.05 was considered to be statistically significant. Data from 798 respondents were included in the analysis. The prevalence of hesitancy toward the second booster of the COVID-19 vaccine was 26.7%. The predictors of second-booster hesitancy were older age (AOR = 1.040, 95 CI = 1.022, 1.058), having received the third dose (first booster) because of instruction by the government (AOR = 2.125, 95% CI = 1.380, 3.274), concern about serious long term side effects of the vaccine (AOR = 4.010, 95% CI = 2.218, 7.250), and opinions of close friends and immediate family members that the booster is harmful (AOR = 2.201, 95% CI = 1.280, 3.785). Conversely, factors that appear to reduce vaccine booster hesitancy were acceptance of the third dose due to the high number of cases and the increasing rate of infection (AOR = 0.548, 95% CI = 0.317, 0.947), the belief that the vaccine will decrease the risk of getting the infection (AOR = 0.491, 95% CI = 0.277, 0.870), and opinions of close friends and immediate family members that the booster is helpful (AOR = 0.479, 95% CI = 0.273, 0.840). In conclusion, more than one-fifth of Malaysians were hesitant to take the second booster of the COVID-19 vaccine. This suggests that appropriate steps that increase vaccine acceptance, taking into consideration the findings of the present study, are needed to address this issue and to foster more positive attitudes toward vaccination. The survey was available in three main languages but limited to people with internet access; hence, it would likely be biased toward younger adults and social media users and exclude those with limited or no internet access, in particular older people. Therefore, the results are not representative of the Malaysian population at large and caution should be exercised when interpreting the findings.

## 1. Introduction

Vaccination is a key public health strategy because it has been shown to be effective in reducing the risk of infection and severe disease [1]. According to the real-time-data shown in COVIDNOW [2], an official national surveillance tool for tracking the progress of COVID-19 vaccination, at least 86% and 84.2% of Malaysians completed the first and second dose of the COVID-19 vaccines, respectively (as of 5 January 2023). On 10 August 2021, the World Health Organization (WHO) agreed with emerging evidence on the need for and timing of an additional vaccine dose of the currently available COVID-19 vaccines for several reasons [3]. Firstly, there is clear evidence on the continual emergence of new COVID-19 variants including those with increased transmissibility and/or virulence; secondly, serum antibody levels in immunized persons will wane over time to levels that are inadequate to prevent or reduce risk of infection from new variants; and thirdly, waning protection continues to adversely affect the health of some risk groups. Based on these considerations/reasons, the decision to add a second booster dose to the full three doses was made and implemented in Malaysia.

Studies on the hesitancy of the general public toward the first booster shot of the COVID-19 vaccine reported levels that spanned a very wide range. These included reports from Japan with the lowest level at 2.1% [4], China with levels that ranged from 6.5% [5] through 8.9% [6] to 23.2% [7], Algeria at 23.8% [8], Italy at 24.7% [9], Poland at 29% [10], Singapore at 30.5% [11], USA from 38.2% [12] to 41.7% [13], India at 44.1% [14], South Korea at 48.8% [15], and lastly Jordan at 55.4% [16].

Overall, the estimated pooled prevalence of hesitancy was 14.3% as reported in a meta-analysis on hesitancy for the first booster of the COVID-19 vaccine [17]. This review stated that the predictors of hesitancy were younger age, low confidence in the COVID-19 vaccine, adverse reactions and discomfort experienced after previous COVID-19 vaccinations, and concerns for serious adverse reactions to COVID-19 booster doses. According to the study from Jordan which reported the highest level of hesitancy (55.4%), the most frequent reasons for the hesitancy were lack of scientific evidence that the booster is beneficial, the duration between the previous dose and the booster being too short, and the opinion that the booster was unnecessary if one had already been infected [16]. In the USA (vaccine hesitancy of 38.2–41.7%), about two-fifths of the people believed that the vaccine was not important for their own health and that of their community, that the vaccine was ineffective and not beneficial, and that vaccine information was not trustworthy [12]. Fear of booster side effects was the major reason for booster hesitancy in the Poland study (hesitancy rate of 29%) [10], while the lack of adequate information regarding the booster dose and not having friends and family members who were diagnosed with COVID-19 infection were found to be predictors of booster hesitancy among the Italian population, where the hesitancy rate stood at 14.3% [9]. The main concern among the people from China (hesitancy rate from 6.5% to 23.2%) was issues related to the safety and effectiveness of the vaccine [6].

Apart from minor and transient adverse reactions to the vaccine, which are accepted by the public, some people may be vulnerable to serious complications following the vaccination for COVID-19. The public have access to information about serious cases of side effects from the news, social media, and social contacts, or may have experienced adverse reactions personally. Previous COVID-19 infection may also affect their perception of the importance of getting the vaccines. As of 5 January 2023, at least 49.7% of Malaysians have received a booster dose. However, it is unknown whether Malaysians received the booster doses due to personal conviction and faith in its effectiveness or were influenced by other external forces. Therefore, the hesitancy toward subsequent booster uptake remains an intriguing question. This question is particularly important to investigate in order to gauge the hesitancy toward the second booster of the COVID-19 vaccine and its associated factors, both of which remain largely unexplored among Malaysians.

Therefore, this study aimed to determine the prevalence and factors associated with hesitancy toward the second dose of booster for the COVID-19 vaccine among Malaysians.

## 2. Materials and Methods

### 2.1. Study Design, Sampling Method and Inclusion Criteria

This was a web-based cross-sectional study involving the general population to examine their hesitancy toward the dose 4 or second booster of the COVID-19 vaccine. We obtained institutional review board approval (Ethical Clearance number: U/SERC/119/2022) from the UTAR Scientific and Ethical Review Committee prior to conducting the data collection (19 August 2022 to 19 November 2022).

Respondents were recruited via social media and telecommunication platforms such as Facebook, Twitter, Instagram, WhatsApp, Telegram, and WeChat. Using the exponential online convenience sampling method, participants were also encouraged to share the link in their network of family members, friends, and colleagues. The inclusion criteria for this study were Malaysians who were at least 18 years old and had completed 3 doses of the COVID-19 vaccine.

### 2.2. Instruments and Variables

This web-based study consists of 12 sections; information on each section is shown in Table S1. We used the Oxford COVID-19 Vaccine Hesitancy Scale, which is a 7-item scale adapted from Freeman et al. [18], with a minor modification by adding “second booster” so that the questions remain relevant for assessing hesitancy toward the second booster of the COVID-19 vaccine. We maintained the scoring method in which the item-specific response options which are coded from 1 (“Definitely”) to 5 (“Definitely not”) were used. A “Don’t know” option is also provided but will be excluded from the scoring. The Cronbach’s alpha of the instrument is 0.97; it has also been shown to be associated with the Vaccine Hesitancy Scale (r = 0.47, *p* < 0.001). The total score could thus range from 7 to 35, where a higher score would indicate a higher level of vaccine hesitancy. For statistical analysis, we did not use the continuous data of the Oxford COVID-19 Vaccine Hesitancy score; instead, we categorized the respondents into two groups (hesitant versus not hesitant). Hesitant is defined as those who endorsed at least one item with a clear vaccine hesitancy response (a response rating of 4 (probably not) or 5 (definitely not)). On the other hand, not hesitant is defined as those who did not endorse any clear vaccine hesitancy response (a response rating of 4 (probably not) or 5 (definitely not)). Nevertheless, the scores of the 7 items were summed to give an aggregate score as shown in the result section. The higher the score on the Oxford COVID-19 Vaccine Hesitancy Scale, the greater the hesitancy toward the booster dose of the COVID-19 vaccine.

### 2.3. Statistical Analysis

The statistical software package IBM SPSS Statistics for Windows Version 21.0 was used for the data analysis. The results of the descriptive analysis were presented either in mean ± SD or n, %. A simple logistic regression was performed to identify variables which were deemed to have association with second-dose hesitancy; variables with a *p*-value < 0.25 in the simple logistic regression were included in the multiple factors logistic regression because a *p*-value set at <0.05 may miss any variables known to be important [19,20]. “Hesitancy on second booster” assessed with the Oxford COVID-19 vaccine was the dependant variable used in the entered model. Any variables with a *p*-value of <0.05 were considered statistically significant.

## 3. Results

Data collection started on 19 August 2022 and ended on 19 November 2022.

### 3.1. Characteristics of Participants

A total of 853 responses were obtained in the study of which 55 were excluded as they did not fulfil the inclusion criteria; thus, 798 responses were retained for data analysis.

Characteristics of the respondents are presented in Table 1. The mean age of respondents was 30.5 years old, with ages ranging from 18 to 76 years. More than half of the respondents were female (64.7%), Chinese (76.1%), had tertiary education (95.6%), and were living in urban areas (86.3%). Regarding personal health issues, only 7.4% had chronic diseases and 6.6% were on medications.

The frequencies of endorsement for each of the Oxford COVID-19 Booster Hesitancy Scale items are summarized in Table S2. This scale manifested excellent reliability with a Cronbach’s alpha value of 0.959 in this cohort of respondents. Overall, the mean value of the Oxford COVID-19 Booster Hesitancy Scale was 17.12 ± 6.53. Out of 798 respondents, 585 (73.3%) had no indication of hesitancy/refusal toward the fourth dose of the vaccine (defined by “Did not endorse any vaccine hesitancy response with a response rating of 4 (probably not) or 5 (definitely not) on any of the 7 items); the remaining 213 respondents (26.7%) indicated that they were clearly hesitant toward or would refuse to take the fourth dose of the vaccine (defined by “Endorsed at least one item with a clear vaccine hesitancy response with a response rating of 4 (probably not) or 5 (definitely not) on any of the 7 items of the scale).

### 3.2. History of COVID-19 Infection and Immunization, and Symptoms after Immunizations

Table 2 depicts the history of COVID-19 infection, immunization, and symptoms after immunization for COVID-19. A significant number (n = 324, 40.6%) of respondents reported a history of COVID-19 infection and the majority (n = 704, 88.2%) of respondents reported they had close friends or immediate family members who were infected. However, only 18.2% (respondents themselves, their close friends, or immediate family members) were hospitalized for the infection.

Regarding COVID-19 immunization, the Sinovac COVID-19 vaccine was the most common first dose of vaccine given to this cohort of respondents (46.5%), followed by Pfizer-BioNTech (34.1%) and AstraZeneca (19.0%). For the second dose, the Sinovac COVID-19 vaccine remains the most common brand at 45.2%, followed by Pfizer-BioNTech (35.6%), AstraZeneca (18.8%), and others (0.4%). For the third dose of the vaccine, Pfizer-BioNTech was the most common (71.2%) brand received by respondents, followed by Sinovac (14.4%) and AstraZeneca (14.3%).

All vaccines can cause mild and temporary reactions, and so do the COVID-19 vaccines. Pain or swelling at the injection site (44.6%) was the most common mild symptom after the first or second dose of immunization, followed by tiredness/weakness (44.4%), fever/chills (32.0%), body/muscle/joint pain (31.8%), and headache (26.3%); other reactions constitute less than 10% each. On the other hand, around one-fifth of respondents (23.7%) did not experience any reactions. Two-thirds (66.8%) of the respondents reported their close friends and immediate family members experienced mild reactions after the first or second dose of the vaccine.

Uncommonly, vaccine recipients could experience serious reactions after the vaccination, and some may lose their lives due to vaccine-induced serious side effects. Details of the results on the events “after 1st or 2nd dose” and “after 3rd dose” are provided in the Table 2. There were very few cases (9 cases or 1.1%) in this cohort of respondents who experienced severe reactions to the first or second dose of vaccines and 5.5% of respondents registered that their close friends or immediate family members had similar severe reactions. Notably, 33 respondents (4.1%) reported they had close friends or immediate family members who died following the first or second dose of the vaccine. Likewise, deaths among close friends and family members were also present following the third dose of COVID-19 vaccines.

### 3.3. Reasons for Accepting the Primary and First Booster Doses of COVID-19 Vaccine and Related Concerns

The reasons for accepting the primary (first 2 doses) and the first booster (third dose) of the COVID-19 vaccine and related concerns were explored.

As shown in Table 3, the top four reasons for accepting the first or second dose were: “I believe that the vaccine will decrease my risk of getting infected” (73.1%), followed by “the number of cases was high/rising” (67.7%), “the number of deaths was high” (45.2%), and “taking them gives me more freedom of movement” (42.7%). The reasons for accepting the third dose of the vaccine remained largely the same, with a decrease in the risk of infection (67.4%) being the most common, followed by the high and increasing number of cases (55.1%), increased freedom of movement (36.6%), and lastly government mandate (36.5%).

Nearly half of the respondents indicated that they were not concerned after receiving the first/second dose of the vaccine (49.1%) and the third dose of the vaccine (44.1%). On the other hand, there were also respondents who were concerned that the COVID-19 vaccines may not fully protect them from infection: 34% in the case of the first and second doses versus 25.7% in the case of the third dose. Concerns about serious long-term side effects were voiced by 29.1% and 27.3% of the respondents with respect to the first/second dose and third dose of vaccines, respectively. Another concern was the relatively short-term protection provided by the vaccine (34.2%).

### 3.4. Reasons for Acceptance and Rejection of the Fourth Dose of the COVID-19 Vaccine

In this cohort of respondents (Table 4), 358 had taken or planned to take the fourth dose of the vaccine and 440 do not plan to take it or remain undecided.

We probed the reasons for accepting the fourth dose of the COVID-19 vaccine among those who had taken or had the interest to be immunized. It was noted that the majority believed that the vaccine will decrease their risk of being infected (80.7%). A significant proportion (42.5%) supported taking the fourth dose, reason being that the “number of cases were high/rising”, and about one fifth (22.1%) intended to take the booster as they perceive themselves to be at high risk of serious illness if infected.

The second subgroup of this cohort comprises 440 respondents who do not plan to take or remained undecided about receiving the fourth dose of the vaccine. The top three reasons endorsed for rejection or hesitancy were limited information to support the effectiveness of the second booster (55%), followed by in decreasing order, the perception that one is already protected by the primary and first booster doses (47.3%), and of being protected due to having been infected (34.3%).

### 3.5. Subjective Opinions and the Sources of Opinions

Table 5 is on the subjective opinions on the booster dose of the COVID-19 vaccine derived from different influential lobbies that the respondents were exposed to. The majority (86.8%) of respondents heard from government sources that taking the booster dose is helpful, while a small proportion (15.8%) claimed that the message from the government is that the booster is neither helpful nor harmful. There are even some, albeit very few (1%), who consider that the message from governmental sources suggests that the booster is harmful.

A different trend was seen in the case of information spread by social media and close friends/immediate family members. Opinions that taking a booster dose is harmful are apparently more commonly shared on social media (17.4%) and among close friends/immediate family members (20.9%). Nevertheless, information that suggests that taking the booster dose is helpful remains the most common message in social media and intimate social groups, which stood at 66.3% and 57.8%, respectively.

### 3.6. Factors Associated with Hesitancy for Dose 4 of the COVID-19 Vaccine

Table 6 shows the factors associated with hesitancy for the second booster of the COVID-19 vaccine. The analysis was performed using the Enter method. The omnibus tests of model coefficients for logistic regression analysis were statistically significant (*p* < 0.05), with a chi-square (X^2^) of 235.369 (*p* < 0.001) in the model. This indicates that the model is better in predicting the outcome than that with the baseline model when only the constant is included. The Nagelkerke (R^2^) was 0.372, which indicates 37.2% of the variation in the outcome could be explained by the independent variables included in the analysis. Moreover, the Hosmer–Lemeshow goodness of fit also showed that the data is fit (*p* must be >0.05) for the model, with X^2^ = 11.041 (*p*-value = 0.199). The model was 81.6% accurate in its prediction of second-booster hesitancy among Malaysians.

Based on the analysis, the predictors of second-booster hesitancy were: older age (AOR = 1.040, 95 CI = 1.022, 1.058), government mandate to take dose 3 as the reason for accepting dose 3 (AOR = 2.125, 95% CI = 1.380, 3.274), concern that dose 3 would cause serious long-term side effects (AOR = 4.010, 95% CI = 2.218, 7.250), and opinions of close friend and immediate family that the booster is harmful (AOR = 2.201, 95% CI = 1.280, 3.785).

We also found some predictors which significantly reduce second-booster hesitancy: the high or increasing number of cases as the reason for accepting dose 3 (AOR = 0.548, 95% CI = 0.317, 0.947), the belief that the vaccine will decrease the risk of getting infection as the reason for accepting dose 3 (AOR = 0.491, 95% CI = 0.277, 0.870), and opinions of close friends and immediate family that the booster is helpful (AOR = 0.479, 95% CI = 0.273, 0.840).

## 4. Discussion

This study aimed to determine the prevalence of and factors associated with hesitancy toward a second booster of the COVID-19 vaccine among the Malaysian general public. This study included various factors that might play an important role in explaining the hesitancy/rejection to receive a second COVID-19 booster shot; these findings could have important public health implications.

### 4.1. Prevalence of Hesitancy towards on Fourth Dose of COVID-19 Vaccine

In this study, the prevalence of hesitancy toward the second booster of the COVID-19 vaccine was 26.7%. To date, there were only two studies on hesitancy on the second booster for the COVID-19 vaccine, one in the USA and the other in Greece (as shown in Table 7). In the study on US citizens, the hesitancy toward the second booster was 66% among the general public who had received an initial booster shot [21], while that among nurses in Greece was 30.9% [22]. The prevalence is considered quite high in both cases, albeit with notable differences which could be due to multiple factors.

Despite the COVID-19 pandemic having been around for more than 3 years (since December 2019), a cumulative death toll of 6.6 million (as of 31 December 2022), and consistently around 2000 deaths daily over months as reported in the Interactive Databases for Coronavirus [23,24], the percentage of those fully vaccinated with primary doses worldwide remains unsatisfactory at 64.4% (as of 31 December 2022). With regards to the booster dose, only 30.4% have received it worldwide. Overall, a greater proportion of the population in Greece and Malaysia were vaccinated with the primary doses (72.2% and 85.1% respectively) and the booster dose (55% and 50.3% respectively) compared with the USA, where only 68.3% and 34.4% were given the primary doses and first booster dose, respectively [23]. A review reported that the overall estimation of booster acceptance rate stood at 81% [25]. Chang et al. [26] reported that the populations from middle-income countries had a satisfactory percentage in willingness to take a booster dose (87.6%), while another review reported that the acceptance rate for the COVID-19 booster dose among low-, middle-, and high-income countries in the Mediterranean regions were 73.4%, 67.9%, and 83.0%, respectively [27].

Concerns for serious reactions is understandably one of the common factors affecting the decision to be vaccinated. A low percentage of those vaccinated experienced severe adverse effects among Malaysians, as reported in two studies (3% for first dose and 4.5% for second dose [28], and 3.4% for first dose and 2.2% for second dose [29]). The result of the present study is similarly low at 1.1% for the first or second dose. This is in sharp contrast to the results of another local study which was also performed via a web-based survey [30]. This study found that moderate to severe side effects (measured using a scoring system) after the primary vaccines was reported by 46.5% of their respondents. We note, however, that the profile of the respondents differs from the present study with respect to the ethnic distribution and infection experience which, together with the different method of measuring the side effects, may partly explain the marked difference. With regards to the first booster vaccine, the Malaysian National Surveillance on Safety of the COVID-19 vaccine gave a value of only 0.1% for severe reaction post-booster [29], whereas our data gives a frequency of 1.6%.

The data from the study on Greeks were mainly from case reports [31,32,33] that examined sporadic cases of severe/odd reactions to the COVID-19 vaccine; there was a lack of observational studies reporting on reactions to the COVID-19 vaccine and its booster. The rate of serious reactions to the COVID-19 vaccine among Americans was 9.2% in those vaccinated with the first/second dose [34], 7.6% of first-booster recipients [35], and 5.1% of second-booster recipients [36]. It is quite clear that the rate of serious reactions to COVID-19 vaccines and the booster among Malaysians was generally much lower than among Americans. This could underlie the much higher level of vaccine hesitancy for the second booster among Americans. However, in actuality, the main reasons expressed by those surveyed were concerns about missing work to get vaccinated and lack of concern about the need for a second booster, underpinning the need to consider cultural differences between people from different geographical regions when comparing the results of different studies.

Other than concerns for serious reactions post-vaccination, another important factor that influences the intention of people with regards to COVID-19 vaccination is national/governmental efforts in promoting vaccinations and building vaccine confidence. In Malaysia, the government engaged social influencers and religious leaders to encourage the populace to register for vaccination and implemented a policy of mandatory vaccination for free mobility, entering business premises, returning to work, and even in border clearance [37]. Further, the Malaysian National Immunization Programme employed many other approaches and actions to promote vaccination, such as active negotiation with multiple vaccine supplier countries/companies for procurement, vaccine prioritization for at-risk people (front liners, those with chronic diseases, and the elderly), and care for the elderly and the vulnerable by providing special services and assistance for easy access to the vaccine. This could explain the relatively high level of vaccination and lower level of hesitancy among Malaysians, which is ascribed to positive attitudes and a high level of trust among them [37,38].

A different approach was used in Greece, whereby each vaccinated 18–25 year-old was rewarded with a 150 EURO pre-paid card for their first COVID-19 vaccine [39]. On the other hand, the elderly would be fined EURO 113 per month if they were not vaccinated or failed to show proof that they had an appointment for one [40]. In comparison, USA governments had more proactive approaches such as launching educational campaigns to gear up the vaccination rate and update information on the COVID-19 vaccine via TV, radio, and print ads; they also collaborated with local authorities to run on-field engagements and tours in rural communities, inclusive of the minorities and marginalized communities, to highlight the importance of getting a booster dose in multiple languages. They also ran pop-up vaccination clinics/or vaccination trucks in regions with lower vaccine uptake [41]. However, despite such efforts, the reported hesitancy for the second booster is unexpectedly high at 66%.

### 4.2. Factors Associated with the Hesitancy towards on Dose 4 of COVID-19 Vaccine

We found that older age is one of the predictors of second-booster hesitancy; this is consistent with the literature on hesitancy for the second dose of the booster [21]. Older age was also found to be one of the predictors of first-booster hesitancy by several studies [4,5,8,9]. However, older age may not a predictor for COVID-19 booster hesitancy for the first dose of the COVID-19 vaccine in the local context. One possible explanation is that older adults may have received their first dose of the vaccine earlier in the vaccine rollout when there was less information available about the vaccine’s safety and efficacy, and thus have been more willing to take a chance on the vaccine. However, as more information became available, some older adults may have become more cautious and hesitant to receive a booster shot. Additionally, older adults may be more concerned about potential side effects or have more underlying health conditions that they believe may increase their risk for adverse effects from the vaccine. Finally, there may be differences in messaging and communication strategies that have been used to promote the first dose of the vaccine versus the booster shot, which may impact vaccine uptake among older adults. Nevertheless, the COVID-19 booster dose is considered to be critical for older adults as they are at the highest risk for serious infection and death from COVID-19 compared with other age groups, more so with the emergence of new virus variants. Vaccination against COVID-19 was provided free of charge in public hospitals and clinics across Malaysia. Nevertheless, practical constraints and mobility problems among the elderly pose a challenge for access to the vaccines in this subgroup.

Trust in the official information provided, or the lack thereof, is another factor that needs consideration. Consistent with the literature [8], we found that this factor was a predictor of hesitancy for the second booster; indeed, those who express a lack of trust in the information had higher odds of hesitating to take the second booster. Reasons underlying the lack of trust among the skeptical populace include contradictory and sometimes confusing information being aired in mainstream media by different sources from the health authorities particularly during the early days of the pandemic [42]. It was further pointed out that while appropriate measures had been implemented over the course of the pandemic [43], during the initial phase, there was a notable lack of appreciation of its severity and thus a failure to institute prompt actions in order to limit the spread of COVID-19, which was reflected by very high daily cases at that time [44]. When a travel ban was finally implemented in March 2020 [45], it was deemed to be late, as by then there were around 100 thousand daily cases, 210 thousand cumulative cases, and over 8 thousand deaths worldwide [46]. Despite these early difficulties, a state election was held in September 2020 which, as expected, led to an exponential increase in infections and deaths in early 2021 [47]. All the above factors, together with other issues such as inconsistent messaging and implementation of actions by the authorities, were considered to be important obstacles to gaining public trust [47,48,49].

Currently, our vaccination rate of the second booster has remained stagnant over several months, due in part to the fear about the safety of the vaccine arising from widespread circulation about this issue on social media platforms. This is evidenced by our results in that two of our predictors for vaccine hesitancy are: (i) concerns about vaccine side effects and (ii) expressed opinions by close friends and immediate family that the vaccine booster is harmful. The fear of severe complications related to the vaccine booster [50] appears to override the judgement for the need of the additional booster, in particular for the at-risk population. It is acknowledged that vaccine-related serious side-effects are real, although the frequency of occurrence is low; nevertheless, this can induce a disproportionate degree of wariness about the booster vaccine, fuelled further by the dialogue on social media [51].

On the other hand, we also found predictors which are associated with low hesitancy toward a second booster of the COVID-19 vaccine. The hesitancy was reduced, tending toward acceptance of the second booster, when the respondents believed: (i) that the number of cases were high/rising and (ii) that the booster can decrease the risk of being infected. These findings are consistent with the literature [6,7,11], as shown in Table S3 [4,5,6,7,8,9,10,11,12,13,14,15,16]. It has been pointed out that the factors that lead to vaccine hesitancy can only be overcome if the public have access to news on current COVID-19 infectivity trends regionally and internationally, and if they have a clear understanding of the nature of the disease and of the role of vaccination [52]. In addition, influence from close friends and immediate family was one of the important factors for the acceptance or hesitancy toward the booster. It has been suggested that routine updating of the scientific evidence on vaccine efficacy and safety on mainstream media and social media could have a positive, albeit subtle, effect [53]. Indeed, vaccine effectiveness is, as expected, directly associated with vaccine acceptance [50]. This underlies the importance of providing reliable and accurate information to the public.

### 4.3. Strengths and Limitations

First, it is worth mentioning that other than basic demographic factors, this study included multiple factors that were deemed to be important with respect to vaccine hesitancy, such as the history of COVID-19 infection and hospitalization, adverse reactions experienced from dose 1/2 of the COVID-19 vaccine, reasons for accepting dose 1/2, and concerns after accepting dose 1/2. Further, we also included factors such as adverse reactions experienced from dose 3 of the COVID-19 vaccine, reasons for accepting dose 3, concerns after accepting dose 3, and lastly, subjective opinions (positive, neutral, negative) and source of the opinions. Information obtained from this study provided an idea of the extent of booster hesitancy and the concerns surrounding this issue. This could provide helpful and holistic information in designing future programs to tackle hesitancy or refusal to take boosters of the COVID-19 vaccine.

Second, data were collected using an online questionnaire available in Malay, Chinese, and English, the three main languages in this country. We believe that this would increase the inclusiveness of the study. However, the mode of the survey could have excluded seniors who do not have access to the internet or have trouble using technology. Hence, the respondents would be biased toward younger adults and those who have access to and actively engage in social media. On top of that, the sample used in the study may have limitations in terms of its size and diversity with respect to demographic characteristics. Therefore, the findings might not reflect the actual attitude and hesitancy toward the second booster of the COVID-19 vaccine among the entire Malaysian population. Hence, the findings should be interpreted with caution.

### 4.4. Implications

Healthcare practitioners and policymakers can use the results of this study to educate the public about the necessity of the second booster of the COVID-19 vaccine in several ways; first, given that older age was found to be a predictor of second-booster hesitancy in the study, healthcare practitioners and policymakers can focus messaging on the importance of the second booster for this age group. This can be done through targeted messaging on social media or through public service announcements.

Second, the study found that concern about serious long-term side effects of the vaccine was a predictor of second-booster hesitancy. Healthcare practitioners and policymakers can address these concerns by emphasizing the safety and effectiveness of the vaccine, and by providing clear and accurate information about the risks and benefits of vaccination.

Third, the study found that the opinions of close friends and immediate family members that the booster is harmful were a predictor of second-booster hesitancy. Healthcare practitioners and policymakers can leverage social networks to spread accurate information about the vaccine and to address misconceptions.

Fourth, healthcare practitioners and policymakers can engage with communities to understand their specific concerns about the vaccine and to tailor messaging to their needs. This can be done through community events, town hall meetings, or other forms of outreach.

Fifth, to address concerns about the internet and technology access, policymakers can ensure that accurate information about the vaccine is widely available and accessible through multiple channels. They can also make it easy for people to get vaccinated by setting up vaccination clinics in convenient locations and providing clear information on how to schedule appointments.

## 5. Conclusions

More than one-fifth of Malaysians in this study expressed hesitancy toward the second booster of the COVID-19 vaccine. Necessary steps should be taken by the government and public health authorities, in line with the current sentiment, to increase the vaccination rate and foster positive responses toward the need for updating vaccinations.

Based on the empirical findings, stakeholders should conduct more vaccine education and communication, provide accurate, more transparent, and easily understandable information on updated scientific evidence of the effectiveness and safety of booster doses of the COVID-19 vaccine and its potential benefits and risks. This can be communicated through multiple channels, such as social media, community forums, and healthcare providers. Through such initiatives, individuals would be empowered to make informed decisions about vaccination, especially among older adults, and thus help alleviate vaccine hesitancy. Further, the public could also be advised about the importance of accurate information in promoting the health of their friends, family, and the community at large. In summary, it is important to adopt a multi-pronged approach that addresses the various reasons for vaccine hesitancy and the underlying concerns through evidence-based strategies.

## Figures and Tables

**Table 1:** Socio-demographic characteristics of respondents (n = 798).

Characteristics	Category	Overall
Age, years	Mean ± SD	30.46 ± 12.68
	Min–Max values	18–76
Sex	Male	282 (35.3)
	Female	516 (64.7)
Ethnicity	Malay	96 (12.0)
	Chinese	607 (76.1)
	Indian	57 (7.1)
	Others	38 (4.8)
Education Level	Primary education or below	3 (0.4)
	Secondary education	32 (4.0)
	Tertiary education	763 (95.6)
Area of Residence	Urban areas	689 (86.3)
	Rural areas	109 (13.7)
States/Territories of Residence	Johor	80 (10.0)
	Kedah	17 (21)
	Kelantan	7 (0.9)
	Kuala Lumpur	115 (14.4)
	Labuan	1 (0.1)
	Melaka	19 (2.4)
	Negeri Sembilan	25 (3.1)
	Pahang	19 (2.4)
	Penang	41 (5.1)
	Perak	38 (4.8)
	Putrajaya	4 (0.5)
	Sabah	25 (3.1)
	Sarawak	25 (3.1)
	Selangor	377 (47.2)
	Terengganu	5 (0.6)
Chronic Diseases	Without	739 (92.6)
	With	59 (7.4)
On medication for chronic diseases	Not on medication	745 (93.4)
	On medication	53 (6.6)
Oxford COVID-19 Booster Hesitancy Scale (Total score ranges from 1–35)	Mean ± SD	17.12 ± 6.53
	Min–Max values	7–35
	Did not endorse any clear vaccine hesitancy response (a response rating of 4 (probably not) or 5 (definitely not)) on any of the 7 items of the scale	585 (73.3)
	Endorsed at least one item with a clear vaccine hesitancy response (a response rating of 4 (probably not) or 5 (definitely not)) on any of the 7 items of the scale	213 (26.7)
	Internal consistency reliability, Cronbach’s alpha value	0.959

Note: Data were presented either in mean ± SD or N (%) or range of minimum and maximum values.

**Table 2:** History of COVID-infection and immunization (n = 798).

Characteristics	Categories or Details	Overall
History of COVID-19 infection–self	I have never been infected	474 (59.4)
	I had been infected	324 (40.6)
History of COVID-19 infection–close friends or immediate family members	They have never been infected	94 (11.8)
	They had been infected	704 (88.2)
History of Hospitalization due to COVID-19 infection–Self, close friends or immediate family members	We have never been hospitalized	653 (81.8)
	We had been hospitalized	145 (18.2)
Brand of the 1st dose of COVID-19 immunization	AstraZeneca	152 (19.0)
	Pfizer-BioNTech	272 (34.1)
	Sinovac	371 (46.5)
	Others	3 (0.4)
Brand of the 2nd dose of COVID-19 immunization	AstraZeneca	150 (18.8)
	Pfizer-BioNTech	284 (35.6)
	Sinovac	361 (45.2)
	Others	3 (0.4)
Mild reactions after 1st or 2nd dose of COVID-19 immunization–Self	Without any reactions	189 (23.7)
	Headache	210 (26.3)
	Pain or swelling at injection site	356 (44.6)
	Body/muscle/joint pain	254 (31.8)
	Nausea/Vomiting/Diarrhoea	26 (3.3)
	Skin rashes	22 (2.8)
	Fever/Chills	255 (32.0)
	Muscle Spasm/Cramp	62 (7.8)
	Tiredness/Weakness	354 (44.4)
Severe reactions after 1st or 2nd dose of COVID-19 immunization–Self	I did not have a severe reaction	789 (98.9)
	I had a severe reaction	9 (1.1)
Mild reactions after 1st or 2nd dose of COVID-19 immunization–Close friends or immediate family members	They did not have mild reactions	265 (33.2)
	They had mild reactions	533 (66.8)
Severe reactions after 1st or 2nd dose of COVID-19 immunization–Close friends or immediate family members	They did not have severe reactions	754 (94.5)
	They had severe reactions	44 (5.5)
Died after 1st or 2nd dose of COVID-19 immunization–Close friends or immediate family members	No one died	765 (95.9)
	Someone died	33 (4.1)
Brand of the 3rd dose of COVID-19 immunization	Astrazeneca	114 (14.3)
	Pfizer-BioNTech	568 (71.2)
	Sinovac	115 (14.4)
	Others	1 (0.1)
Mild reactions after 3rd dose of COVID-19 immunization–Self	I did not have mild reactions	396 (49.6)
	I had mild reactions	402 (50.4)
Severe reactions after 3rd dose of COVID-19 immunization–Self	I did not have severe reactions	785 (98.4)
	I had severe reactions	13 (1.6)
Mild reactions after 3rd dose of COVID-19 immunization–Close friends or immediate family members	They did not have mild reactions	340 (42.6)
	They had mild reactions	458 (57.4)
Severe reactions after 3rd dose of COVID-19 immunization–Close friends or immediate family members	They did not have severe reactions	763 (95.6)
	They had severe reactions	35 (4.4)
Died after 3rd dose of COVID-19 immunization–Close friends or immediate family members	No one died	778 (97.5)
	Someone died	20 (2.5)

Note: Data were presented in n (%).

**Table 3:** Reasons for accepting the primary and first booster doses of COVID-19 vaccine and concerns after immunization (n = 798).

	Descriptions	1st or 2nd Dose	3rd Dose
Reasons for accepting COVID-19 immunizations	I believe that the vaccine will decrease my risk of getting infected	583 (73.1)	538 (67.4)
	The number of cases were high/rising	540 (67.7)	440 (55.1)
	The number of deaths were high	361 (45.2)	260 (32.6)
	Taking them gives me more freedom of movement	341 (42.7)	292 (36.6)
	The government told us to take them	315 (39.5)	291 (36.5)
	My doctor/friends/family advised me to take them	180 (22.6)	167 (20.9)
	Most people I know were taking them	166 (20.8)	164 (20.6)
	I am at high risk of serious illness if I get infected	146 (18.3)	149 (18.7)
	My employer required me to take them	138 (17.3)	128 (16.0)
Concerns after receiving COVID-19 immunizations	I was not concerned	392 (49.1)	352 (44.1)
	I was concerned that they may not protect me fully from the infection	271 (34.0)	205 (25.7)
	I was concerned that they may cause serious long-term side effects	232 (29.1)	218 (27.3)
	I was concerned that its protection effects will not last that long	NIL	273 (34.2)

Note: Data were presented in n (%).

**Table 4:** Reasons for accepting the fourth dose (second booster) of COVID-19 vaccine and concerns (n = 798).

Reasons and Concerns	Descriptions	Taken and Plan to Take 4th Dose (n = 358)	Do not Plan to Take or Undecided (n = 440)
Reasons for accepting the fourth dose	I believe that the vaccine will decrease my risk of getting infected	289 (80.7)	NIL
	The number of cases were high/rising	152 (42.5)	NIL
	I am at high risk of serious illness if I get infected	79 (22.1)	NIL
	Government told us to take them	59 (16.5)	NIL
	My doctor/friend/family advised me to take them	43 (12.0)	NIL
	Most people I know were taking them	36 (10.1)	NIL
	My employer required me to take them	29 (8.1)	NIL
Reason for rejecting the fourth dose	I am waiting for more data to support its usefulness	NIL	242 (55.0)
	I am unsure of its effectiveness in preventing the infection	NIL	208 (47.3)
	I am already protected by the third dose and/or by having been infected already	NIL	151 (34.3)
	I have concerns about the side effects due to my own experience from the vaccination, experience of my friends and family, and reports from social media	NIL	140 (31.8)
	It could cause serious long-term side effects	NIL	118 (26.8)
	The protection does not last long	NIL	77 (17.5)
	I have no risk or very low risk of infection	NIL	47 (10.7)
	I have no time	NIL	31 (7.0)
	I do not trust COVID-19 vaccines	NIL	18 (4.1)
	I do not trust the type of vaccine offered free of charge	NIL	6 (1.4)
	I do not believe in vaccines in general	NIL	2 (0.5)
What is your preference for 4th dose of vaccine	AstraZeneca	46 (12.8)	NIL
	Cansino-Bio	1 (0.3)	NIL
	Pfizer-BioNTech	258 (72.1)	NIL
	Sinovac	45 (12.6)	NIL
	Others	8 (2.2)	NIL

Note: Data were presented in n (%).

**Table 5:** Subjective opinions expressed by Government, social media, and close friends/immediate family members on booster doses for the COVID-19 vaccine (n = 798).

	Taking Booster Dose Is Helpful	It Is Neither Helpful or Harmful	Taking Booster Dose Is Harmful
Government opinions on booster dose	693 (86.8)	126 (15.8)	8 (1.0)
Social media opinions on booster dose	529 (66.3)	294 (36.8)	139 (17.4)
Friend/Family opinions on booster dose	461 (57.8)	357 (44.7)	167(20.9)

**Table 6:** Predictors of hesitancy toward the second booster of the COVID-19 vaccine (n = 798) using simple and multiple factors logistic regression analysis.

Factors	Category	Simple Logistic Regressions			Multiple Factors Logistic Regressions		
		COR	95 CI	*p*-Values	AOR	95% CI	*p*-Values
Age	It is a continuous data	1.034	1.022, 1.046	<0.001	1.040	1.022, 1.058	<0.001
Sex	Female (Reference)	-	-	-	-	-	-
	Male	0.681	0.485, 0.957	0.027	0.862	0.566, 1.315	0.491
Ethnicity	Malay and Chinese (Reference)	-	-	-			
	Indian and Others	1.721	1.097, 2.699	0.018	0.885	0.494, 1.585	0.681
Education	Primary school/None (Reference)	-	-	-	-	-	-
	Secondary school	1.048	0.085, 12.876	0.971	-	-	-
	University/College or above	0.715	0.065, 7.931	0.785	-	-	-
Area of residence	Living in Urban (Reference)	-	-	-	-	-	-
	Living in Rural	1.228	0.789, 1.911	0.363	-	-	-
Chronic diseases	Without chronic disease (Reference)	-	-	-	-	-	-
	With chronic disease	1.451	0.826, 2.549	0.196	0.629	0.130, 3.040	0.564
On medication	Not on medication (Reference)	-	-	-	-	-	-
	On medication	1.587	0.884, 2.848	0.122	0.792	0.151, 4.164	0.783
History of COVID-19 infection (self)	I have never been infected (Reference)	-	-	-	-	-	-
	I was infected before	1.098	0.798, 1.509	0.566	-	-	-
History of COVID-19 infection (close friends or immediate family)	They have never been infected (Reference)	-	-	-	-	-	-
	They were infected before	1.141	0.693, 1.879	0.604	-	-	-
History of hospital admission due to COVID-19 infection (self, close friends or immediate family)	We have never been admitted (Reference)	-	-	-	-	-	-
	We were admitted before	0.849	0.559, 1.289	0.443	-	-	-
Mild reaction after Dose 1 or 2 (self)	Without any mild reaction (Reference)	-	-	-	-	-	-
	With mild reaction	1.421	0.964, 2.095	0.076	1.469	0.909, 2.375	0.116
Severe reaction after Dose 1 or 2 (self)	Without any severe reaction (Reference)	-	-	-	-	-	-
	With severe reaction	1.379	0.342, 5.562	0.652	-	-	-
Mild reaction after Dose 1 or 2 (Close friends or immediate family)	Without any mild reaction (Reference)	-	-	-	-	-	-
	With mild reaction	1.661	1.168, 2.362	0.005	1.300	0.809, 2.089	0.279
Severe reaction after Dose 1 or 2 (Close friends or immediate family)	Without any severe reaction (Reference)	-	-	-	-	-	-
	With severe reaction	1.452	0.763, 2.766	0.256	-	-	-
Died due to Dose 1 or 2 (Close friends or immediate family)	No one died (Reference)	-	-	-	-	-	-
	Someone died	1.603	0.775, 3.318	0.203	0.488	0.147, 1.617	0.240
Reason of accepting Dose 1 or 2 (1)	No (Reference)	-	-	-	-	-	-
	The number of cases were high/rising	0.632	0.456, 0.877	0.006	1.011	0.601, 1.700	0.967
Reason of accepting Dose 1 or 2 (2)	No (Reference)	-	-	-	-	-	-
	The number of deaths were high	0.870	0.634, 1.194	0.389	-	-	-
Reason of accepting Dose 1 or 2 (3)	No (Reference)	-	-	-	-	-	-
	I am at high risk of serious illness if I get infected	0.802	0.527, 1.222	0.304	-	-	-
Reason of accepting Dose 1 or 2 (4)	No (Reference)	-	-	-	-	-	-
	I believe that the vaccine will decrease my risk of getting infection	0.371	0.265, 0.520	<0.001	0.725	0.399, 1.320	0.293
Reason of accepting Dose 1 or 2 (5)	No (Reference)	-	-	-	-	-	-
	Most people I know were taking them	0.595	0.390, 0.908	0.016	0.609	0.324, 1.114	0.123
Reason of accepting Dose 1 or 2 (6)	No (Reference)	-	-	-	-	-	-
	The government told us to take them	1.202	0.874, 1.653	0.257	-	-	-
Reason of accepting Dose 1 or 2 (7)	No (Reference)	-	-	-	-	-	-
	My doctor/friends/family advised me to take them	0.796	0.540, 1.172	0.248	0.856	0.511, 1.435	0.556
Reason of accepting Dose 1 or 2 (8)	No (Reference)	-	-	-	-	-	-
	My employer required me to take them	1.305	0.874, 1.947	0.193	0.679	0.338, 1.364	0.276
Reason of accepting Dose 1 or 2 (9)	No (Reference)	-	-	-	-	-	-
	Taking them gives me more freedom of movement	1.330	0.971, 1.824	0.076	1.240	0.743, 2.067	0.410
Concern that the dose 1 or 2 will not fully protected me	No (Reference)	-	-	-	-	-	-
	Yes	1.141	0.822, 1.585	0.431	-	-	-
Concern that the dose 1 or 2 would cause serious long term side effect	No (Reference)	-	-	-	-	-	-
	Yes	3.125	2.243, 4.355	<0.001	0.865	0.480, 1.559	0.630
Mild reaction after Dose 3 (self)	Without any mild reaction (Reference)	-	-	-	-	-	-
	With mild reaction	1.157	0.845, 1.585	0.362	-	-	-
Severe reaction after Dose 3 (self)	Without any severe reaction (Reference)	-	-	-	-	-	-
	With severe reaction	2.393	0.795, 7.204	0.121	2.675	0.600, 11.931	0.197
Mild reaction after Dose 3 (Close friends or immediate family)	Without any mild reaction (Reference)	-	-	-	-	-	-
	With mild reaction	1.524	1.101, 2.109	0.011	0.780	0.499, 1.220	0.276
Severe reaction after Dose 3 (Close friends or immediate family)	Without any severe reaction (Reference)	-	-	-	-	-	-
	With severe reaction	2.732	1.381, 5.406	0.004	1.637	0.516, 5.191	0.402
Died due to Dose 3 (Close friends or immediate family)	No one died (Reference)	-	-	-	-	-	-
	Someone died	2.833	1.162, 6.904	0.022	1.398	0.274, 7.128	0.687
Reason of accepting Dose 3 (1)	No (Reference)	-	-	-	-	-	-
	The number of cases were high/rising	0.546	0.398, 0.749	<0.001	0.548	0.317, 0.947	0.031
Reason of accepting Dose 3 (2)	No (Reference)	-	-	-	-	-	-
	The number of deaths were high	0.778	0.553, 1.097	0.152	1.130	0.665, 1.918	0.651
Reason of accepting Dose 3 (3)	No (Reference)	-	-	-	-	-	-
	I’m at high risk of serious illness if I get infected	0.850	0.562, 1.284	0.439	-	-	-
Reason of accepting Dose 3 (4)	No (Reference)	-	-	-	-	-	-
	I believe that the vaccine will decrease my risk of getting infection	0.341	0.246, 0.473	<0.001	0.491	0.277, 0.870	0.015
Reason of accepting Dose 3 (5)	No (Reference)	-	-	-	-	-	-
	Most people I know were taking them	0.635	0.418, 0.966	0.034	0.851	0.468, 1.547	0.596
Reason of accepting Dose 3 (6)	No (Reference)	-	-	-	-	-	-
	The government told us to take them	1.731	1.257, 2.383	0.001	2.125	1.380, 3.274	0.001
Reason of accepting Dose 3 (7)	No (Reference)	-	-	-	-	-	-
	My doctor/friends/family advised me to take them	1.097	0.750, 1.606	0.633	-	-	-
Reason of accepting Dose 3 (8)	No (Reference)	-	-	-	-	-	-
	My employer required me to take them	1.553	1.036, 2.326	0.033	1.460	0.715, 2.978	0.298
Reason of accepting Dose 3 (9)	No (Reference)	-	-	-	-	-	-
	Taking them gives me more freedom of movement	1.425	1.034, 1.964	0.030	1.028	0.605, 1.745	0.919
Concern that the dose 3 will not fully protected me	No (Reference)	-	-	-	-	-	-
	Yes	1.079	0.756, 1.540	0.676	-	-	-
Concern that the dose 3 protective effect not last long	No (Reference)	-	-	-	-	-	-
	Yes	1.441	1.042, 1.993	0.027	1.468	0.965, 2.234	0.073
Concern that the dose 3 would cause serious long term side effect	No (Reference)	-	-	-	-	-	-
	Yes	4.577	3.258, 6.429	<0.001	4.010	2.218, 7.250	<0.001
Government opinion on dose 3 (1)	No (Reference)	-	-	-	-	-	-
	Taking booster dose is helpful	0.567	0.369, 0.873	0.010	1.147	0.341, 3.857	0.828
Government opinion on dose 3 (2)	No (Reference)	-	-	-	-	-	-
	It is neither helpful of harmful	1.465	0.973, 2.205	0.067	1.331	0.443, 3.994	0.610
Government opinion on dose 3 (3)	No (Reference)	-	-	-	-	-	-
	Taking booster is harmful	8.449	1.692, 42.192	0.009	4.538	0.411, 50.128	0.217
Social media opinion on dose 3 (1)	No (Reference)	-	-	-	-	-	-
	Taking booster dose is helpful	0.447	0.323, 0.617	<0.001	0.910	0.468, 1.770	0.781
Social media opinion on dose 3 (2)	No (Reference)	-	-	-	-	-	-
	It is neither helpful of harmful	1.521	1.104, 2.094	0.010	0.782	0.404, 1.514	0.466
Social media opinion on dose 3 (3)	No (Reference)	-	-	-	-	-	-
	Taking booster is harmful	3.525	2.410, 5.157	<0.001	1.839	0.998, 3.390	0.051
Friends and family opinion on dose 3 (1)	No (Reference)	-	-	-	-	-	-
	Taking booster dose is helpful	0.379	0.275, 0.523	<0.001	0.479	0.273, 0.840	0.010
Friends and family opinion on dose 3 (2)	No (Reference)	-	-	-	-	-	-
	It is neither helpful of harmful	1.663	1.213, 2.281	0.002	0.950	0.528, 1.709	0.864
Friends and family opinion on dose 3 (3)	No (Reference)	-	-	-	-	-	-
	Taking booster is harmful	3.808	2.658, 5.455	<0.001	2.201	1.280, 3.785	0.004

Note: Multiple factor logistic regression was analyzed using the Enter Method.

**Table 7:** Summary of literature findings on hesitancy toward the second booster of the COVID-19 vaccine.

Studies	Prevalence, %	Population	Predictors of Hesitancy
Motta [21]	66	General public in USA	Concern about missing work to vax Not convinced the booster is necessary Ideology of conservatism Older age above 65 years old
Galanis [22]	30.9	Nurses in Greece	Low educational level Married status Absence of chronic disease Good/very good self-perceived physical health Lack of flu vaccination during 2021 Frontline nurses who provided healthcare services to COVID-19 patients during the pandemic Nurses who had not been diagnosed with COVID-19 during the pandemic Those with at least one relative/friend who has died from COVID-19 Increased compliance with hygiene measures Increased fear of a second booster dose COVID-19 vaccine Decreased trust in the COVID-19 vaccine

## Data Availability

The data underlying this article are available in the article and in its online Supplementary Material.

## Supplementary Materials

The following supporting information can be downloaded at: https://www.mdpi.com/article/10.3390/vaccines11030638/s1, Table S1: Description of sections in the online survey on second booster hesitancy; Table S2: The frequency of endorsement for the 7 items of the Oxford COVID-19 Booster Hesitancy Scale (n = 798); Table S3: Summary of findings on factors associated with intention towards the first booster of the COVID-19 Vaccine.
