# Supplementary material for: COVID-19 Vaccine Booster Hesitancy in Malaysia: A Web-Based Cross-Sectional Study
Source: Vaccines (Basel). 2023 Mar 13;11(3):638. doi: 10.3390/vaccines11030638 (PMC10058917; doi:10.3390/vaccines11030638)
Supplement: Supplementary file 1 [file vaccines-11-00638-s001.zip › vaccines-2232075-supplementary.pdf]

Table S1. Description of sections in the online survey on Second booster Hesitancy.

Sections	Details	Remarks
1. Demographics	• Age<br>• Sex<br>• Ethnicity<br>• Education level<br>• Area of Residence<br>• State/Territories of Residence	
2. History of chronic disease	• Chronic diseases<br>• On medication for chronic diseases	
3. History of COVID-19 infection and hospitalization	• Personal<br>• Close friend and immediate family	
4. Adverse reaction experienced from Dose 1/2 of COVID-19 vaccine	• Personal<br>• Close friend and immediate family	
5. Reasons for accepting Dose 1/2 of COVID-19 vaccine	• 9 items	Self-formulating
6. Concerns after accepting Dose 1/2 of COVID-19 vaccine	• 3 items	Self-formulating
7. Adverse reaction experienced from Dose 3 of COVID-19 vaccine	• Personal<br>• Close friend and immediate family	
8. Reasons for accepting Dose 3 of COVID-19 vaccine	• 9 items	Self-formulating
9. Concerns after accepting Dose 3 of COVID-19 vaccine	• 4 items	Self-formulating
10. Status of Dose 4 of COVID-19 vaccine	• Preference of brand for dose 4<br>• Reasons of accepting dose 4 (7 items)<br>• Reasons of rejecting dose 4 (11 items)	Self-formulating
11. Subjective opinions (Positive, neutral, negative) and source of opinions	• Government<br>• Social media<br>• Close friend and immediate family	Self-formulating
12. Oxford COVID-19 Vaccine Hesitancy Scale	• 7 items	With minor modification

Table S2: The frequency of endorsement for the 7 items of the Oxford COVID-19 Booster Hesitancy Scale (n=798).

Oxford COVID-19 Booster Hesitancy Scale	1 mark	2 marks	3 marks	4 marks	5 marks
13. When the 4th dose of COVID-19 vaccine was made available for registration in MySejahtera, I will<br>• 1 mark: definitely take it<br>• 5 marks: definitely will not take it)	250 (31.3)	189 (23.7)	231 (28.9)	83 (10.4)	45 (5.6)
• When the 4th dose of COVID-19 vaccine is easily available (in walk-in vaccination centres, I<br>• 1 mark: will get it as soon as possible<br>• 5 marks: will refuse to get it	180 (22.6)	286 (35.8)	191 (23.9)	86 (10.8)	55 (6.9)
14. I would describe my attitude towards receiving the 4th dose of COVID-19 vaccine as<br>• 1 mark: very positive (supportive)<br>• 5 marks: totally negative (against it)	176 (22.1)	189 (23.7)	343 (43.0)	61 (7.6)	29 (3.6)
15. If the 4th dose of COVID-19 vaccine was available at my local clinic, I would<br>• 1 mark: get it as soon as possible<br>• 5 marks: never get it	182 (22.8)	337 (42.2)	161 (20.2)	73 (9.1)	45 (5.6)
2. If my friends/family were thinking of getting 4th dose of COVID-19 vaccine, I would<br>• 1 mark: strongly encourage them<br>• 5 marks: suggest that they do not get the booster dose	140 (17.5)	240 (30.1)	349 (43.7)	39 (4.9)	30 (3.8)
3. I would describe myself as<br>• 1 mark: eager to get the 4th dose of COVID-19 vaccine<br>• 5 marks: anti-booster for COVID-19	90 (11.3)	334 (41.9)	256 (32.1)	110 (13.8)	8 (1.0)
4. Taking the 4th dose of COVID-19 vaccine is<br>• 1 mark: absolutely important<br>• 5 marks: absolutely not important	111 (13.9)	248 (31.1)	351 (44.0)	68 (8.5)	20 (2.5)
Note: Data was presented in n (%).					

Table S3: Summary of Findings on Factors associated with Intention towards the first booster of COVID-19 Vaccine.

Study	Country	Hesitancy rate, %	Predictors of hesitancy
Yoshida et al., [4]	Japan	2.1	Factors associated with hesitancy:<br>• Higher antibody level (AOR = 2.52; 95% CI: 1.27–4.99)
Wang et al., [5]	China	6.5	Factors associated with hesitancy:<br>• Unemployment (adjusted odds ratio [AOR] 2.428, 95% CI: 1.590–3.670)<br>• Low monthly income (AOR 2.854,95% CI: 1.561–5.281)
Tung et al., [6]	China	8.9	*Factors associated with acceptance:<br>• Confidence in the safety of the COVID-19 vaccines (OR = 3.20)<br>• Confidence in the effectiveness of the COVID-19 vaccines against SARS-CoV-2 (OR = 2.25)<br>• Confidence in the effectiveness of the COVID-19 vaccine against its variants (useful versus [vs.] useless, OR = 2.33),<br>• Previous vaccine recipients (OR = 3.09)
Wu et al., [7]	China	23.2	Factors associated with hesitancy:<br>• High perceived severity (adjusted odds ratio [AOR] = 0.69)<br>• Response cost (AOR = 0.47)<br>*Factors associated with acceptance<br>• high perceived susceptibility (AOR = 1.19)<br>• Response efficacy (AOR = 2.13)<br>• Self-efficacy (AOR = 3.33)
Lounis et al., [8]	Algeria	23.8	Factors associated with hesitancy:<br>• Females<br>• Older age<br>• High school education<br>• Those with diabetes or hypertension<br>• With history of COVID-19 infection
Folcarelli et al., [9]	Italy	24.7	Factors associated with hesitancy:<br>• Older (ACSE: 1.03)<br>• Females (ACSE: 1.23)<br>• Staying alone (ACSE: -0.99)<br>• No need more additional information regarding the booster dose of the COVID-19 vaccine (ACSE: 0.46)<br>• Higher self-rated health status after second dose of COVID-19 vaccine (ACSE: 1.22)<br>• Having received information regarding the booster dose of the COVID-19 vaccine from official government organization (ACSE: 1.68)<br>• Having friends or family members who were diagnosed with COVID-19 (ACSE: 2.0)
Rzymski et al., [10]	Poland	29.0	NA
Tan et al., [11]	Singapore	30.5	Factors associated with hesitancy:<br>• No formal education/Primary education (AOR: 0.109)<br>• Secondary education (AOR: 0.540)<br>• Perceived benefit (AOR 0.257)<br>• Necessity for booster vaccine (AOR: 0.547)<br>• Perceived concerns (AOR: 1.698)<br>• Perceived risk of COVI-19 (AOR:0.79)
Yadete et al., [12]	USA	38.2	Factors associated with hesitancy:<br>• Low functional literacy<br>• Low communicative literacy<br>• Low critical literacy<br>• High school education and below
Batra et al., [13]	USA	41.7	Factors associated with hesitancy:<br>• Low perceived advantages of vaccine

			• High perceived disadvantages of vaccine<br>• Low behavior confidence<br>• Low in adaptation to physical environment
Achrekar et al., [14]	India	44.1	Factors associated with hesitancy:<br>• Older age<br>• Male<br>• High education<br>• Rural<br>• Living with vulnerable population<br>• Family/friends tested positive COVID-19<br>• Poor vaccine confidence<br>• Poor vaccine literacy
Noh et al., [15]	South Korea	48.8	Factors associated with booster hesitancy:<br>• Women (OR, 1.25; 95% CI, 1.05 to 1.50)<br>• Younger people (OR, 1.44; 95% CI, 1.17 to 1.77)<br>• Those with a lower education level (OR, 2.05; 95% CI, 1.10 to 3.82)<br>• Those who received the mRNA-1273 vaccine type (OR, 2.01; 95% CI, 1.65 to 2.45)<br>• Those who experienced serious adverse events following previous COVID-19 vaccination (OR, 2.03; 95% CI, 1.47 to 2.80)
Al-Qerem et al., [16]	Jordan	55.4	Factors associated with booster hesitancy<br>• Had mild or no symptoms due to COVID-19 vaccine (AOR: 0.54)<br>• I took it because of the imposed law, not because I believe it (AOR: 20.88)<br>• I took it out of conviction and because of laws (AOR: 2.68)<br>• Low Self-perceived risk (AOR: 1.7)
Note: Factors associated with first booster acceptance.<br>ACSE – Adjusted coefficient Standard errors<br>AOR – Adjust odd ratio<br>OR – odd ratio			
